# Electrochemical Oxidation of Methanol and Small Polyols in Neutral Media: The Effect of the Interfacial pH on Dynamic Instabilities

**DOI:** 10.1021/acsomega.5c10287

**Published:** 2025-12-16

**Authors:** Nayara Gomes dos Santos, Evaldo Batista Carneiro-Neto, Lauren Moreti, Rafael Luiz Romano, Fabio Henrique Barros Lima, Ernesto Chaves Pereira, Elton Sitta

**Affiliations:** † Department of Chemistry, 67828 Federal University of Sao Carlos, Rod. Washington Luis, km 235, ZIP, 13565-905 Sao Carlos, São Paulo, Brazil; ‡ São Carlos Institute of Chemistry (IQSC), University of São Paulo (USP), ZIP, 13560-970 São Carlos, São Paulo, Brazil

## Abstract

The electrochemical oxidation of alcohols and polyols is interesting to feed direct alcohol fuel cells or to provide both protons and electrons to H_2_ production in electrolyzers. While these small organic molecules are oxidized in a wide pH range, oscillatory phenomena are commonly observed only in acidic media, and the phenomenon is restricted for few molecules in neutral or alkaline media. Herein, the electrochemical oxidation of methanol, ethylene glycol, and glycerol was studied in unbuffered Na_2_SO_4_ solutions (pH 8.2) by means of cyclic voltammetry, chronoamperometry, and chronopotentiometry. Changes of the near-surface solution pH (NSSpH) were estimated by mathematical modeling, revealing changes up to 6.5 pH units and a 4 mm depletion layer after 100 s of polarization. NSSpH changes provide an acidic environment to alcohol/polyol oxidation as observed by means of the oscillations features. Facilitating the mass-transport condition, NSSpH becomes close to the bulk, in which both the low potential activity and the potential oscillations are no longer observed. Applying mathematical modeling to the galvanostatic timeseries, it was possible to evaluate the changes in NSSpH and to conclude that the observed oscillations are due to the pH decrease triggered by the alcohol/polyol oxidation.

## Graphical abstract

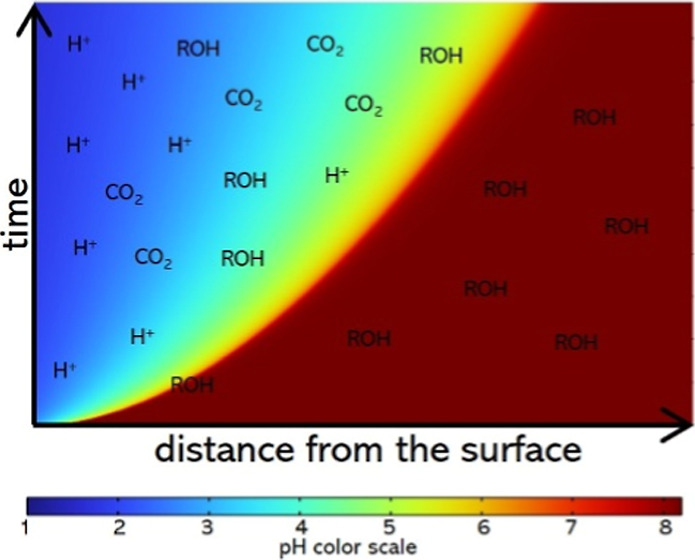

## 1 Introduction

The electrocatalysis of small organic molecules oxidation reactions has been studied in the last decades aiming to feed energy-conversion devices.^1,2^ In this context, several small carbon chain molecules such as alcohols/polyols, aldehydes/ketones, and carboxylic acids could provide electrons and protons to allow oxygen reduction in a fuel cell or H_2_ electrosynthesis in electrolyzers. While these systems were widely studied in acidic or alkaline media, fewer works are dedicated to understanding the electrocatalytic features near pH 7.

During the electrochemical oxidation of small organic molecules, while the electrons flow to current collectors, protons are delivered to the solution side of the interface. The bulk pH remains unchanged during electrochemical processes; however, some pH changes near the electrodes are expected, especially in quiescent solutions. Therefore, the so-called near-surface solution pH (NSSpH) takes into account the pH changes up to a few tens of μm from the electrode surface to the solution. Mundy et al.^3^ employing a rotating ring-disc electrode setup observed cathodic currents at the ring electrode due to H^+^ reduction provided by methanol oxidation at the disc electrode in Na_2_SO_4_ solution (pH 4.9). Interestingly, the authors observed mirrored profiles for the ring and the disc cyclic voltammograms, indicating the couple of electron and proton production. A similar conclusion was obtained by Figueiredo et al.^4^ but for ethanol oxidation in alkaline media. Recently, Nagita et al.^5^ developed a finite element-based model able to describe pH changes in porous electrodes during acidic water electrolysis. The results revealed that the pH can exceed 7 in the cathodes working at current levels similar to those employed in electrolyzers. In neutral media, the scenario is much more complex because, while the pH changes fast with proton accumulation in unbuffered solutions, the impact of the distinct anions in buffered solutions needs to be considered.

The electrochemical oxidation of small organic molecules can also undergo dynamic instabilities such as oscillations, the systems being classified as HN-NDR oscillators in which a N-shaped negative differential resistance (NDR) is partially hidden (H) by the presence of adsorbed reaction residues,^6^ e.g., CO_ad_. In practice, the oscillation phenomenon can be understood as the competition between at least two feedback loops, one being fast and responsible to increase the potential and the other(s) being responsible for restoring the potential for lower values. The latter is commonly associated with the oxidation of the adsorbed reaction residues at a high potential. The presence of oscillations and their features such as frequency, amplitude, and shape is extremely dependent on the interface conditions, making the oscillations an interesting tool to follow changes on the interface.

Melle et al.^7^ studied the solution pH effect on oscillations during methanol oxidation, concluding that the oscillations are restricted to pH < 3.50 in buffered hydrogen-phosphate solutions. Fiori et al.^8^ described oscillations in a similar system but in a NaF/HClO_4_ (pH 5.6) solution. Interestingly, there are no reports about oscillation during methanol electrochemical oxidation at Pt in pH higher than 6 in buffered solutions.

On the other hand, ethylene glycol (EG) and glycerol electro-oxidation reactions in Pt can undergo oscillatory behavior in both acidic and alkaline media.^9–16^ The oscillations in alkaline media display a myriad of complex patterns,^11,13^ and near the Hopf bifurcation, these oscillations tend to have a higher frequency than those observed in acidic media for similar alcohol concentrations. Moreover, no oscillatory behavior was found during the EG oxidation reaction in the 4–13 pH range.^10^ Finally, mass-transport plays an important role in oscillatory behavior.^7,15,16^ For instance, oscillations during the glycerol oxidation reaction in alkaline media tend to cease, increasing the mass-transport, the effect being strongly dependent on the cations in the supporting electrolyte, e.g., Li^+^, Na^+^, and K^+^.^16^ The glycerol reaction pathways can be tailored through mass-transport due to soluble active intermediates concentration change or interfacial pH maintenance.^7,16^ These small changes on the interface due to the intermediate’s accumulations are sufficient to trigger or to cease the oscillations; therefore, this phenomenon can be employed as a powerful tool to analyze the interface along electrochemical processes.

NSSpH is likely to change during oscillations timeseries in a quiescent solution due to the proton accumulation; however, this feature is slightly explored or discussed. On the other hand, this parameter becomes extremely important to understand oscillations features such as its onset or extinguishment in a timeseries, as well as pattern changes along the timeseries, especially in neutral solutions.

In this study, we investigated the electro-oxidation of methanol, ethylene glycol, and glycerol in unbuffered Na_2_SO_4_ solutions near pH 7. We take advantage of some cyclic voltammetry features to estimate NSSpH by means of numerical simulations. Moreover, oscillatory behavior was studied both at quiescent solutions and under mass-diffusion control, the distinct features being explained by the NSSpH changes estimated by our numeral model.

## 2 Experimental Procedures

The experiments were conducted in three distinct electrochemical cells employed for (i) oscillation characterization, (ii) studies controlling mass-transport with a rotating disc electrode (RDE), and (iii) online Electrochemical Mass Spectrometry (EC-MS) analysis. Regardless of the cell, the supporting electrolyte consisted of 0.5 mol L^–1^ Na_2_SO_4_ with the pH adjusted by neutralization of 0.5 mol L^–1^ NaOH solution (prepared from dissolution of NaOH Metal grade, 99.9 from Sigma-Aldrich) with H_2_SO_4_ (Suprapur, Mallinckrodt). After the electrolyte purge in the electrochemical cell, the pH was 8.2 ± 0.2. All of the solutions were prepared with ultrapure 18.2 MΩ cm^–1^ water. Ag/AgCl/KCl_sat_ with a double Luggin served as the reference electrode, and a high-area Pt grid served as the counter electrode. The electrolyte was purged with Argon (5.0 N White-Martins), and during the experiment, this gas was maintained on the cell’s headspace. For the oscillation’s characterization, a mirror-like Pt flag (0.54 cm^2^) was employed as the working electrode (WE). The electrode was flame-annealed before each experiment and cooled in the cell’s headspace. Once the electrode area is small, the overall measured currents are lower than 2 mA, which implies an ohmic drop lower than 20 mV, considering 10 Ω of solution resistance as affeered by electrochemical impedance spectroscopy. Therefore, the available data was not corrected by an ohmic drop. The RDE studies were performed with a Pt disc (5 mm diameter) embedded in Teflon. Before the experiment, the surface was polished with diamond paste up to 0.25 μm and rinsed in ethanol and water. Online EC-MS analysis was performed using an OmniStar gas analyzer equipped with a stainless-steel capillary probe. The assembly of the experimental setup was done in a similar way to that described in ref 17. Briefly, a disc-shaped platinum mesh (WE, 10 mm diameter) followed by three PTFE membranes (Gore-Tex, 50 μm thickness and 0.02 μm pore size) was inserted into a PEEK holder, which was positioned at the interface between the mass spectrometer and the electrochemical cell. For electrical contact, a platinum wire was inserted between the PEEK holder and the WE. The geometric area of the WE exposed to the electrolyte was 0.38 cm^2^. During the experiments, the ionic currents of *m*/*z* (mass/charge) fragments 2 (H_2_) and 44 (CO_2_) were recorded versus time and applied potential.

The electrochemical cells were cleaned in KMnO_4_ alkaline solution for at least 12 h followed by H_2_O_2_/acidic solution and rinsed several times in boiling water. The cleaning protocol is detailed in ref 18. An Autolab Potentiostat/Galvanostat (PGSTAT128) equipped with the SCAN250 modulus controlled the applied potential or the current during the experiments.

Despite the complexity of the alcohols’ electrooxidation mechanism, to perform the numerical simulation, only two semireactions were considered: alcohol electrooxidation (r1) yielding 1:1 electron/proton ratio and hydrogen evolution reaction (r2):

r1 $$ROH\to RO+nH^{+}+ne^{-}$$

r2 $$2H^{+}+2e^{-}⇌H_{2}$$

Then, the first reaction releases protons at the electrode vicinity, decreasing NSSpH, and the second reaction consumes the protons to produce dissolved hydrogen gas, which can be further oxidized to restore the protons at the interface.

Additionally, the concentration of protons is related to the concentration of hydroxyl ions according to the water autoionization reaction:

r3 $$H_{2}O⇌H^{+}+OH^{-}$$

For simplicity, methanol transport was disregarded since its concentration is at least 100 times greater than the concentrations of other species, even considering their local variations. As a fully supported electrolyte was assumed, migration transport of the electroactive species can be neglected; then, only the diffusion and convection transport of H^+^, OH^–^, and H_2_ were considered. They obey the Nernst–Planck equation:^19^

1 $$\frac{\partial c_{i}}{\partial t}=D_{i}\frac{\partial^{2}c_{i}}{\partial x^{2}}+0.51023\omega^{3/2}\nu^{-1/2}x^{2}\frac{\partial c_{i}}{\partial x}+R_{i}$$

in which *c*_*i*_, *D*_*i*_, and *R*_*i*_ are the instantaneous local concentration, the diffusion coefficient, and the homogeneous reaction term of the specie *i*, respectively, with *i* being H^+^, OH^–^, or H_2_. ω is the angular frequency and ν is the kinematic viscosity. For H^+^ and OH^–^, *R*_*i*_ describes the water autoionization expressed in r3, and it is written as *k*_f_(*K*_W_ – *c*_H^+^_ *c*_OH^–^_), where *k*_f_ is the rate constant of water association reaction, *K*_W_ is the water autoionization equilibrium constant, and *c*_H^+^_ and *c*_OH^–^_ are instantaneous local concentrations of H^+^ and OH^–^, respectively. For H_2_, *R*_*i*_ is zero because it does not take part in any homogeneous reaction.

At the beginning, the concentrations of H^+^ and OH^–^ are given by the initial pH measured experimentally, which is 8.2, and the H_2_ is considered as initially absent. The increase of protons at the interface occurs by the alcohol/polyol oxidation according to r1; therefore, the experimental charge produce in a 106.3 s polarization was considered to compute the proton increase. Introducing the experimental charge (by means of experimental current, *j*_exp_, integration) in the simulation allowed us to consider complex steps during the oxidation of the organic molecules, such as surface poisoning, mechanistic changes with pH, etc. The electrode was polarized at 0.500 V (vs Ag/AgCl/KCl) for 100 s followed by a negative going scan at 0.100 V s^–1^ from 0.500 V to −0.700 V, and the region of positive current (100 s at 0.500 V + cyclic voltammogram from 0.500 to −0.058 V) was considered to estimate the number of protons delivered. After the step including the experimental parameters, the simulation followed the negative going scan from −0.058 to −0.700 V, considering both residual currents provided by alcohol oxidation *j*_r1_ and the dynamics of proton reduction oxidation, *j*_r2_.

The faradaic current density for step r1 during the negative going scan for the −0.059 and −0.700 V range is given by a Tafel equation:^20^

2 $$j_{r1}=j_{0,r1}10^{\left(E_{app}-E_{eq,r1}\right)/A}$$

in which *j*_0,*i*_ and *E*_eq,*r*1_ are, in this order, the exchange current density and equilibrium potential of reaction *r*1, respectively, *E*_app_ is the applied potential, and *A* is the anodic Tafel slope.

The faradaic current density for step *r*2 is given by the Butler–Volmer equation:

3 $$j_{r2}=j_{0,r2}\left\{\left[\frac{c_{H_{2}}\left(0,t\right)}{c_{ref}}\right]e^{\left[\alpha F\left(E_{app}-E_{eq,r2}\right)/RT\right]}-{\left[\frac{c_{H^{+}}\left(0,t\right)}{c_{ref}}\right]}^{2}e^{\left[-\left(2-\alpha \right)F\left(E_{app}-E_{eq,r2}\right)/RT\right]}\right\}$$

in which *j*_0,*r*2_ and *E*_eq,*r*2_ are, in this order, the exchange current density and equilibrium potential of reaction r2, *c*_H_2__(0,*t*) and *c*_H^+^_(0,*t*) are the instantaneous concentrations of the electroactive species at the electrode interface, *c*_ref_ is a reference concentration chosen to be 1.0 mol L^–1^, α is the transfer coefficient, *F* is the Faraday constant, *R* is the ideal gas constant, and *T* is the absolute temperature.

Additionally, the capacitive local current density, *j*_C_, is considered in eq 4:

4 $$j_{C}=C_{dl}\frac{\partial E_{app}}{\partial t}$$

in which *C*_dl_ is the capacitance of the double layer. The total current density, *j*_T_, is the sum of *j*_*r*1_, *j*_*r*2_, and *j*_C_, which once multiplied by the area of the electrode yields the total current.

If the electrode is stagnant (ω = 0), an efficiency factor was introduced to make it possible for the height of the peaks in the simulation to be similar to those obtained experimentally, as stated in eq 5. It is important to note that the major contribution to the Faradaic current at low potential comes from *j*_r2_ once (*E*_app_ – *E*_eq,*r*1_) is negative and the exponential part of eq 2 goes to zero.

5 $$j_{r1}+j_{r2}+j_{C}=\{\begin{array}{ll} \omega =0, & eff\times j_{\exp}\\ \omega >0, & j_{\exp} \end{array}$$

The boundary conditions at the electrode surface connect the surface concentrations with the faradaic current density contribution that flows externally, as follows:

6 $$D_{H^{+}}{\left(\frac{\partial c_{H^{+}}}{\partial x}\right)}_{x=0}=-\frac{\left(j_{r1}+j_{r2}\right)}{F}$$

7 $$D_{H_{2}}{\left(\frac{\partial c_{H_{2}}}{\partial x}\right)}_{x=0}=\frac{1}{2}\frac{j_{r2}}{F}$$

As the electrode is large enough (i.e., ∼0.2 cm^2^), the edge effects can be disregarded, and the simulation was performed in a 1D geometry, whose length is about 5.8 mm (this distance is defined by the expression $6\sqrt{D_{H^{+}}\tau }$ , with *D*_H^+^_ being the diffusion coefficient of H^+^ in 0.930 mol L^–1^ methanol in water, 7.52 × 10^–5^ cm^2^ s^–1^,^21^ and τ being the total simulated time, which is 124.65 s).

The parameters are summarized in Table 1.

**Table 1:** Summary of Parameters Used in the Finite Element Simulations

symbol	value	description
pH^0^	8.2^a^	initial/bulk pH
$c_{H^{+}}^{0}$	0.0063 μmol L^–1^ ^a^	initial/bulk concentration of H^+^
$c_{{OH}^{-}}^{0}$	1.585 μmol L^–1^ ^a^	initial/bulk concentration of OH^–^
*V*	0.100 V s^–1^ ^a^	scan rate
*C*_dl_	20 μF cm^–2^ ^b^	capacitance of the double layer
*c*_ref_	1 mol L^–1^ ^b^	reference concentration
α	0.5^b^	transfer coefficient
*D*_H^+^_	7.52 × 10^–5^ cm^2^ s^–1^ ^c^	diffusion coefficient of H^+^
*D*_OH^–^_	4.29 × 10^–5^ cm^2^ s^–1^ ^c^	diffusion coefficient of OH^–^
*D*_H_2__	4.13 × 10^–5^ cm^2^ s^–1^ ^c^	diffusion coefficient of H_2_
ν	1.11 × 10^–2^ cm^2^ s^–1^ ^21^	kinematic viscosity of 0.930 mol L–1 H_3_COH in H_2_O
*k*_f_	1.41 × 10^11^ L mol^–1^ s^–1^ ^19^	rate constant of water association reaction
*K*_W_	1.00 × 10^–14^ ^19^	water equilibria
*j*_0,*r*1_	16.85 mA cm^–2^ ^a^	exchange current density of step *r*1
*E*_eq,*r*1_	1.108 V^a^	reference equilibrium potential of step *r*1
*A*	0.268 V^a^	anodic Tafel slope of step *r*1
*j*_0,*r*2_	10 A cm^–2^ ^d^	reference exchange current density of step *r*2
*E*_eq,*r*2_	–0.335 V^a^	reference equilibrium potential of step *r*2
Eff	34%^a^	efficiency factor assumed only for the first step of the simulation and when the solution is stagnant

a Value chosen based on the experimental conditions.

b Value arbitrarily chosen.

c Estimated based on the Stokes–Einstein equation, which states that the diffusion coefficient is inversely proportional to the solvent viscosity (*D*∼1/η), from their values in water, with η­(0.930 mol L^–1^ H_3_COH in H_2_O) = 1.100 mPa s and η­(H_2_O) = 0.890 mPa s.^21^

d Value optimized by simulation to match the experimental data.

The equations were numerically solved using the finite element method (FEM) in COMSOL Multiphysics 6.0. The mesh was built defining a minimum element size of 10 nm at the electrode boundary, from where they slowly grow until reaching the maximum element size of 50 μm. The entire mesh consists of 1000 elements. The simulations were performed on a personal computer with a processor Intel Core I7-7800X with 6 cores working at 3.50 GHz and 128 GB of RAM memory (5 min).

## 3 Results and Discussion

### 3.1 Experimental Electrochemical Analysis

Figure 1 shows Pt flag cyclic voltammograms at 0.10 V s^–1^ in 0.5 mol L^–1^ Na_2_SO_4_ and 1.0 mol L^–1^ methanol (a), ethylene glycol (b), or glycerol (c). Regardless of the organic molecule, in the potential range from 0.0 to 1.0 V, it is possible to recognize two regions of oxidation processes during the positive going scan and one in the negative going scan, the latter usually called the reactivation peak. These features agree with those reported in the literature for pH ≤ 7.^10,13,22^

**Figure 1:**
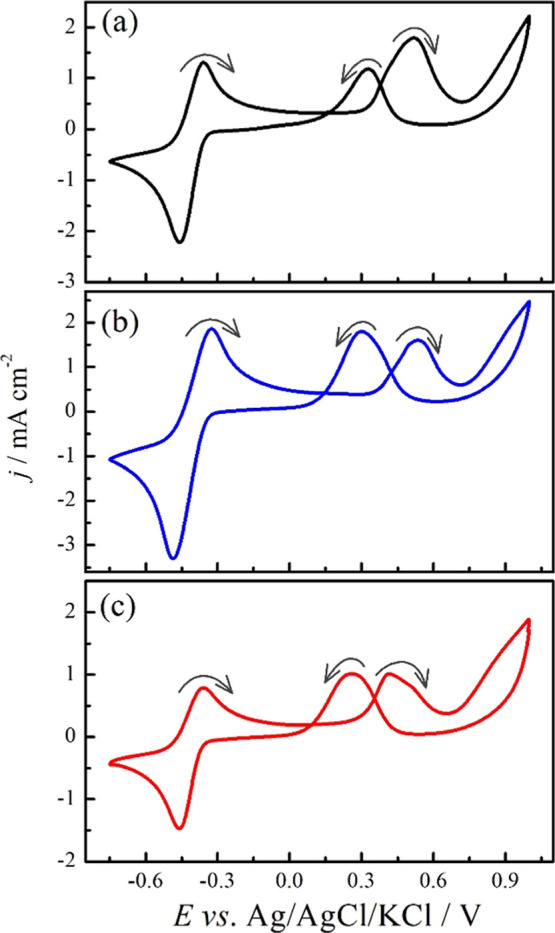
Pt cyclic voltammograms (5th cycle) at 0.10 V s^–1^ in Na_2_SO_4_ (0.5 mol L^–1^, pH 8.2 ± 0.2) and methanol (a), ethylene glycol (b), and glycerol (c). The arrows indicate the sweep directions.

At a potential lower than 0.0 V, a redox pair is clearly observed for the three systems. Interestingly, the anodic process is not observed during the first cycle, and the cathodic current is only observed after the potential execution for high values. Both anodic and cathodic processes increase with the cycles (see the Supporting Information files for methanol oxidation). Controlling the mass-transport by means of the RDE system, it can be observed that while the processes occurring at high potential are slightly affected by the mass-transport, the redox peak at low potential is extinguished (see the Supporting Information files). In a quiescent solution, the cyclic voltammogram profiles in the RDE system fully agree with those shown in Figure 1. We addressed this redox feature to the H^+^/H_2_ pair with H^+^ being formed during the alcohol/polyol’s oxidation processes, for instance, for the total oxidation to CO_2_ or yielding aldehydes/ketones and carboxylic acids. The presence of carboxylic acids could act as a H^+^ source; however, as will be revealed by computer simulations, NSSpH reaches values lower than the p*K*_a_ of formic acid (3.7), glycolic acid (3.8), and glyceric acid (3.5).

The applied potential was based on the Ag/AgCl/KCl reference system, which can be converted to a reversible hydrogen electrode (RHE) using eq 8:

8 $$E_{RHE}=E_{Ag\left|AgCl\right|{KCl}_{sat}}+E_{Ag\left|AgCl\right|{KCl}_{sat}}^{0}+\left(0.0592\times pH\right)$$

in which *E*_RHE_ and $E_{Ag\left|AgCl\right|{KCl}_{sat}}$ are the potentials in the RHE and Ag|AgCl|KCl_sat_ scales, respectively, and $E_{Ag\left|AgCl\right|{KCl}_{sat}}^{0}$ is the standard potential for the Ag|AgCl|KCl_sat_ equilibrium.

The solutions have a pH 8.2 ± 0.5 as measured in an electrochemical cell. Therefore, the cyclic voltammograms took place from −0.06 to 1.68 V vs RHE, and the *E*_1/2_ for the low-potential redox pair is at 0.28 V vs RHE for the three systems. These values are higher than expected for H^+^/H_2_ equilibrium in these experimental conditions. On the other hand, the presence of H^+^ from alcohol oxidation shifts the pH to lower values; therefore, it is expected that the potential in the RHE scale is lower than the above-mentioned values, and their value is estimated in the Computational Simulations section.

To confirm the presence of H_2_, EC-MS experiments were conducted in the Pt/Na_2_SO_4_-methanol system (Figure 2). The Faradaic current (*i*_F_) vs *E* (Figure 2a) is similar to that observed in Figure 1 excepting for the oxidation current of redox pair occurring at low potential, probably by the diffusion of species produced in the cathodic part. The ionic current from *m*/*z* = 2, corresponding to H_2_, clearly increases in the low potential region, indicating that the cathodic current observed relates to H_2_ production. There is experimental evidence of NSSpH changes due to methanol oxidation. The conclusion can be extended to both EG and glycerol due to the similar redox features. The presence of CO_2_ during methanol oxidation is also confirmed by the presence of the *m*/*z* = 44 signal, following the Faradaic current during the positive going scan which attests to the synchronization between Faradaic and ionic currents. The presence of CO_2_ also indicates that methanol oxidation can deliver up to 6 electrons per molecule, but it is also possible that other reaction pathways are happening, yielding formaldehyde and formic acid.

**Figure 2:**
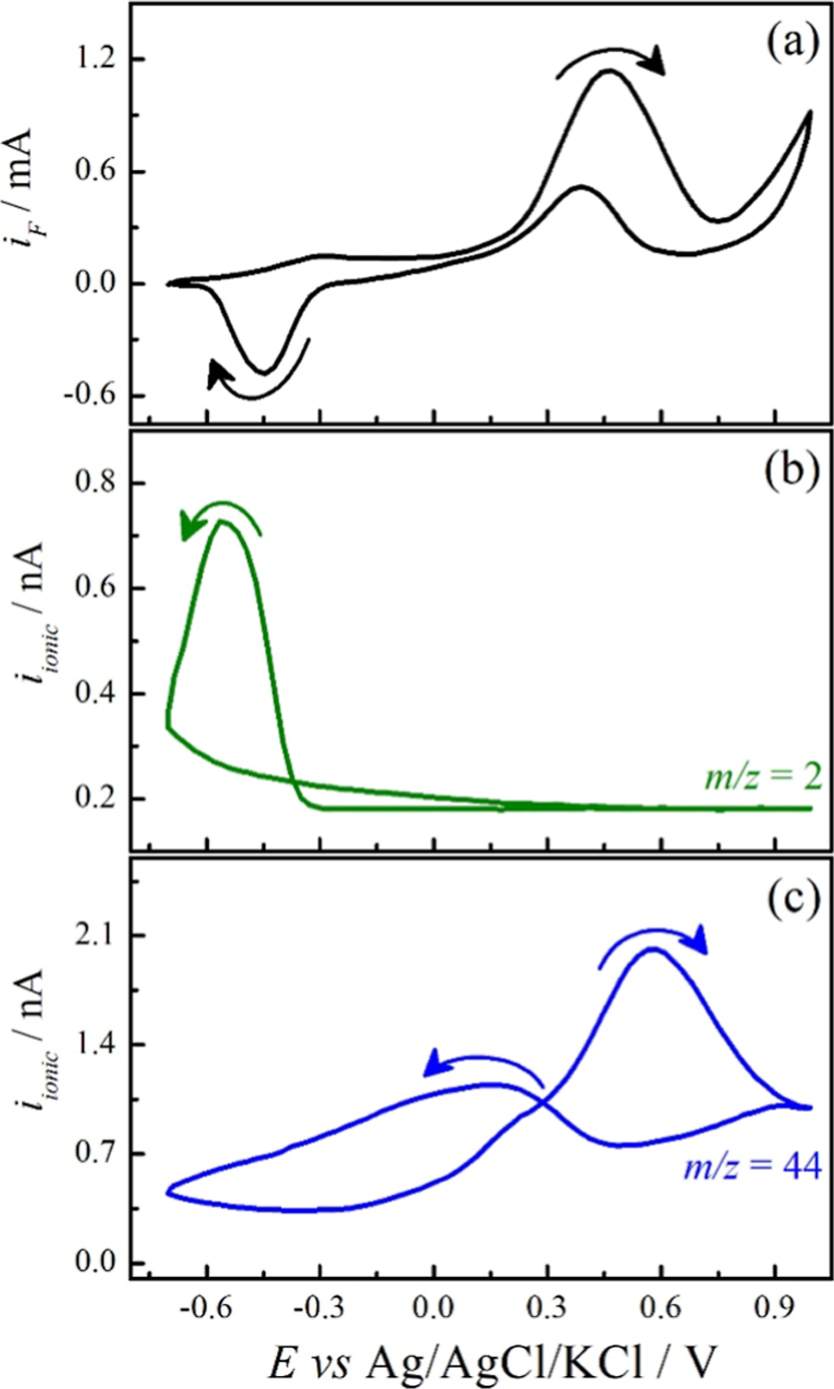
Faradaic (*i*_F_) (a) and ionic (*i*_ionic_) currents of *m*/*z* 2 (b) and 44 (c) fragments obtained during EC-MS measurement of Pt cycliv voltammetry at 0.01 V s^–1^. Electrolyte: Na_2_SO_4_ (0.5 mol L^–1^, pH 8.2 ± 0.2) and methanol (1.0 mol L^–1^).

Galvanostatic timeseries were collected for the systems described in Figure 1. Oscillations in electrochemical systems are very sensitive to the interface conditions; therefore, to map oscillatory behavior, we employed the Pt band in which it is possible to perform the flame annealing activation protocol and to compare the results with our previous works.^8,16,22^ The applied current values corresponded to 50, 25, 12.5, and 6.25% of the maximum current peak at ca. 0.5 V for each cyclic voltammogram in Figure 1. All of the timeseries collected can be found in the Supporting Information files, and the representative timeseries with potential oscillations are shown in Figure 3.

**Figure 3:**
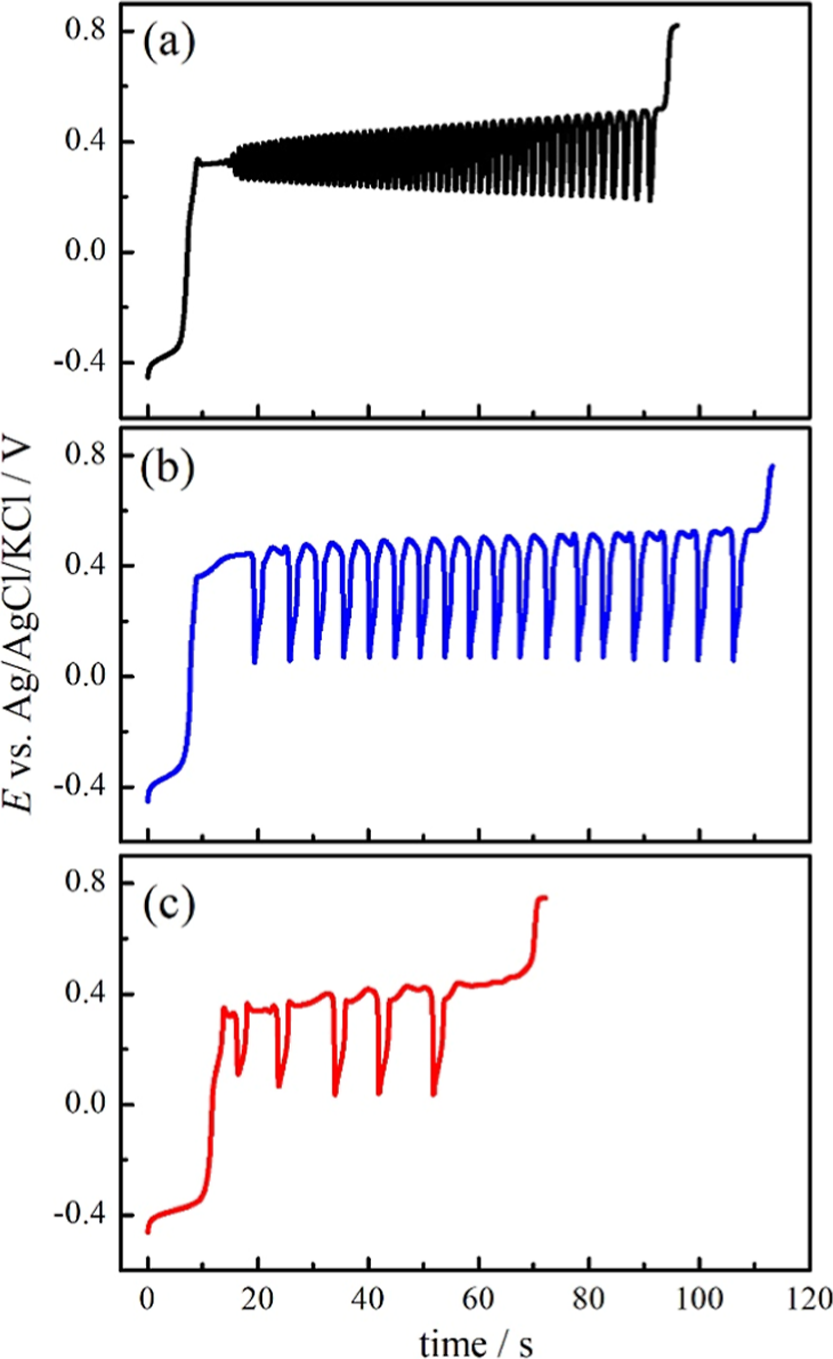
Representative galvanostatic timeseries for methanol (a), ethylene glycol (b), and glycerol (c). The applied currents were 0.46, 0.39, and 0.31 mA cm^–2^, respectively.

The potential oscillations occur from ca. 0.00 to 0.50 V vs Ag/AgCl/KCl and end with the potential increase to values higher than 0.70 V. In the presence of methanol, Period 1 oscillations are observed for a wide current range; however, in the presence of EG or glycerol, period 2 and/or mixed mode patterns are observed for all the studied currents. Considering only the Period 1 oscillations in each timeseries, Figure 4a shows the frequencies (ω) in function of normalized time, i.e., 0 and 1, correspond to the first and last Period 1 oscillations observed in each timeseries. The mean values are also listed in Table 2.

**Figure 4:**
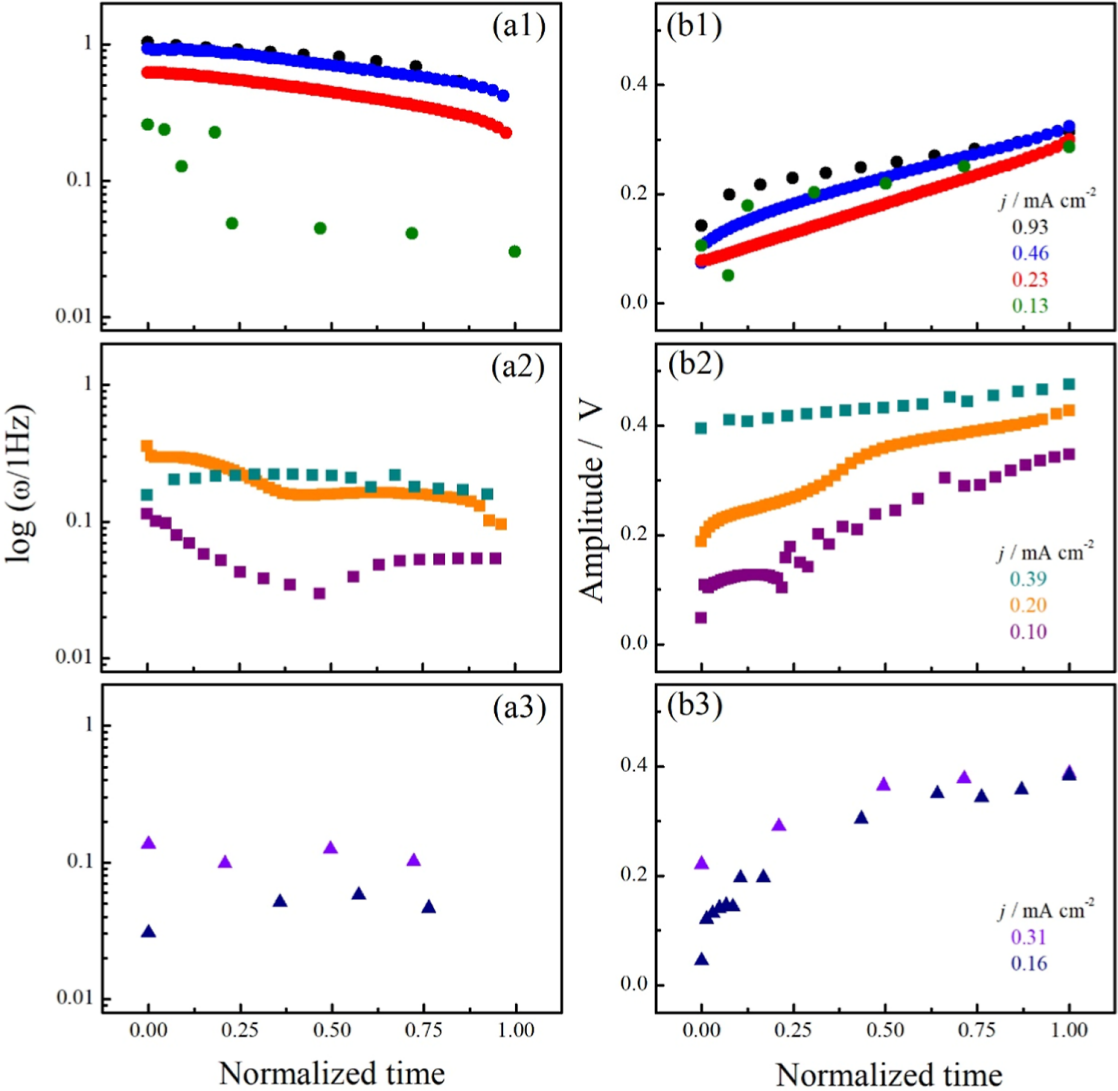
Period 1 oscillations frequencies (ω) (a) and amplitude (b) extract from galvanostatic timeseries in the presence of methanol (a(1), b(1)), EG (a(2), b(2)), or glycerol (a(3), b(3)). Details in the text and timeseries are in the Supporting Information files.

**Table 2:** Mean Frequencies (ω_mean_) for Period 1 Oscillations at Applied Current Densities

active species	*j*/mA cm^–2^	ω_mean_/Hz
methanol	0.13	0.13 ± 0.10
	0.23	0.47 ± 0.11
	0.46	0.74 ± 0.15
	0.93	0.83 ± 0.15
EG	0.10	0.06 ± 0.02
	0.20	0.20 ± 0.02
	0.39	0.21 ± 0.06
glycerol	0.16	0.05 ± 0.01
	0.31	0.11 ± 0.02

Regardless of the active species, ω increases with current, the mean values following the sequence ω_mean,methanol_ > ω_mean,EG_ > ω_mean,glycerol_ at comparable current densities. Moreover, these oscillations frequencies are comparable with those reported in the literature for acidic media.^10,13,22^ Figure 4b brings the amplitude of the oscillations (*E*_max_ – *E*_min_) for the whole timeseries (including the non-Period 1 oscillations). While the amplitudes for oscillations in the presence of methanol are up to 300 mV, they are up to 400 mV for the systems with EG or glycerol, and in some condition to the EG system reaching even superior values. The poisoning/freeing rates of surface during oscillations were estimated by the d*E*/d*t* values at constant current^23^ and shown in the function of the potential in the Supporting Information files. The values are slightly higher than those observed for methanol^8,22^ and EG^24^ in acidic media; however, they are lower than the values obtained in alkaline media during the oscillatory EG electro-oxidation reaction.

As pointed out in the Introduction section, there is a lack of literature reports about the presence of oscillations in neutral media during methanol, EG, or glycerol electrooxidation reactions. Herein, the oscillations have similar patterns and ω_mean_ of those described for the acidic media, which can be connected to the pH shift in the interface. To quantify the effect of NSSpH on the electrooxidation processes, in the next section, we have estimated the H^+^ accumulation from alcohol oxidation by means of computer simulations.

### 3.2 Computer Simulations

The first step of the simulation connected the experimental current recorded at 0.5 V for 106.3 s with the release of protons at the electrode interface, in which only for the stagnant condition an efficiency factor of 34% (eq 5) was assumed to ensure that the simulated peak height matches those obtained experimentally cyclic voltammogram profile, as discussed below. Figure 5 shows NSSpH calculated during the first step of the simulation, which used the experimental current as the boundary condition to calculate the concentration gradients for all the considered species in solution.

**Figure 5:**
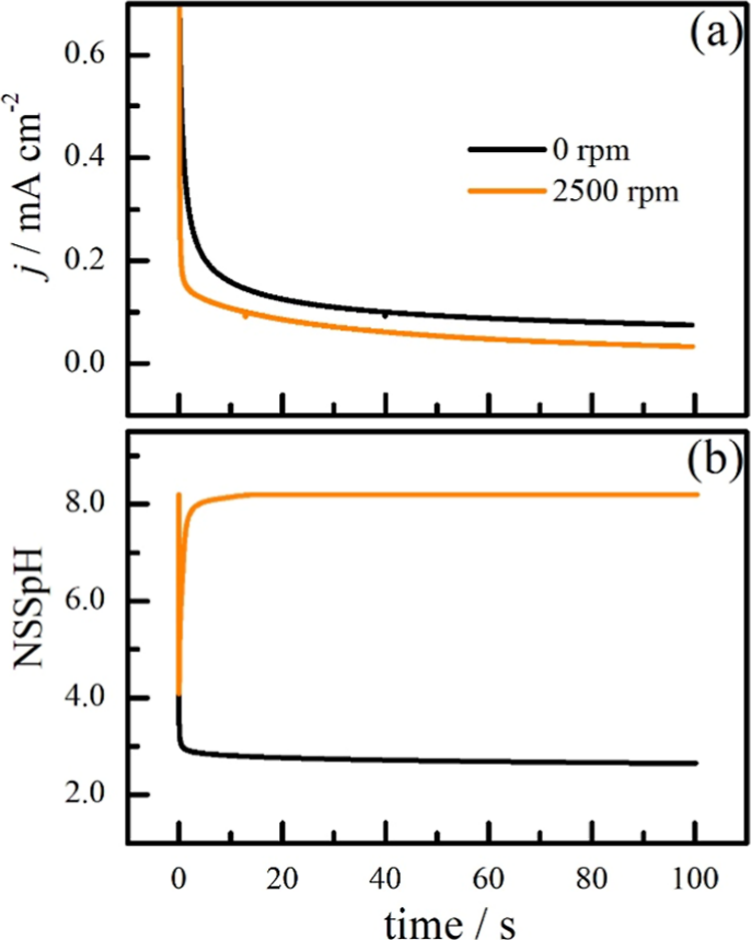
Experimental currents at 0.5 V vs Ag/AgCl/KCl_sat_ (a) and NSSpH estimated by simulation (b) for a stagnant (back line) and for a 2500 rpm rotating disc electrode (orange line). Other experimental conditions are the same as Figure 1.

In the absence of the convective flow (0 rpm), NSSpH quickly decreases at the beginning of polarization, and its value was kept close to 2.7 after 100 s due to the balance established between the generation of protons at the interface and the mass-transport by diffusion that carried them away from the electrode. However, for the case in which there was transport by convection (2500 rpm), at the beginning of the polarization, the local pH decreased to about 4, but as the convective transport is more effective, it spread all the generated protons, keeping NSSpH close to the bulk pH. Interestingly, the curves at 2500 rpm depict lower current than the curve at 0 rpm. It is important to highlight that in these experimental conditions, methanol electro-oxidation reaction is not limited by mass-diffusion; therefore, the changes are probably due to the effect of NSSpH on the reaction, in agreement with ref 22.

Figure 6 compares the experimental and simulated voltammetric currents obtained after the polarization described in Figure 5, i.e., the initial NSSpH is the same as that at *t* = 100 s. In the Figure 6 right axis (blue), NSSpH was estimated by simulation during the cyclic voltammetry.

**Figure 6:**
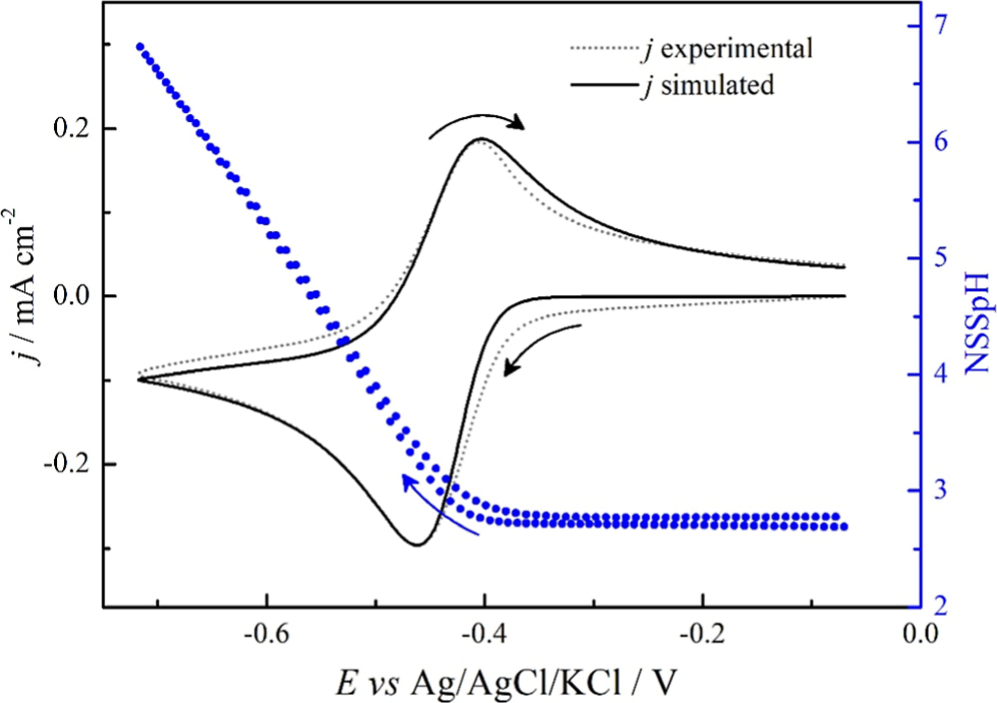
Comparison between the experimental (dotted line) and simulated (solid line) voltammetric currents and the near-surface solution pH (NSSpH) estimated by simulation (blue line).

The redox pair experimentally observed is successfully described by the model in terms of the potential range, with the current peak being adjusted by the 0.34 efficiency factor. The protons from alcohol oxidation remain close to the electrode surface and, during the negative-going scan, they were reduced to H_2_, which was assumed to be fully dissolved in the solution (no bubble formation). In this process, the local pH increased, almost reaching the neutral value. When the sweep direction was reversed, the dissolved H_2_ is oxidized to H^+^, decreasing the NSSpH again. The H^+^ reduction followed by the H_2_ oxidation takes place in the potential region of the redox pair that appears after the first voltammetric cycle in the presence of alcohols (Figure 1).

The model was also applied to follow NSSpH changes at constant current, which was the condition in which the oscillations were observed experimentally. Assuming a faradaic efficiency of 100%, a 0.46 mA cm^–2^ was applied as boundary conditions in the simulation for a time of 100 s. The spatiotemporal evolution of the local pH is shown in Figure 7.

**Figure 7:**
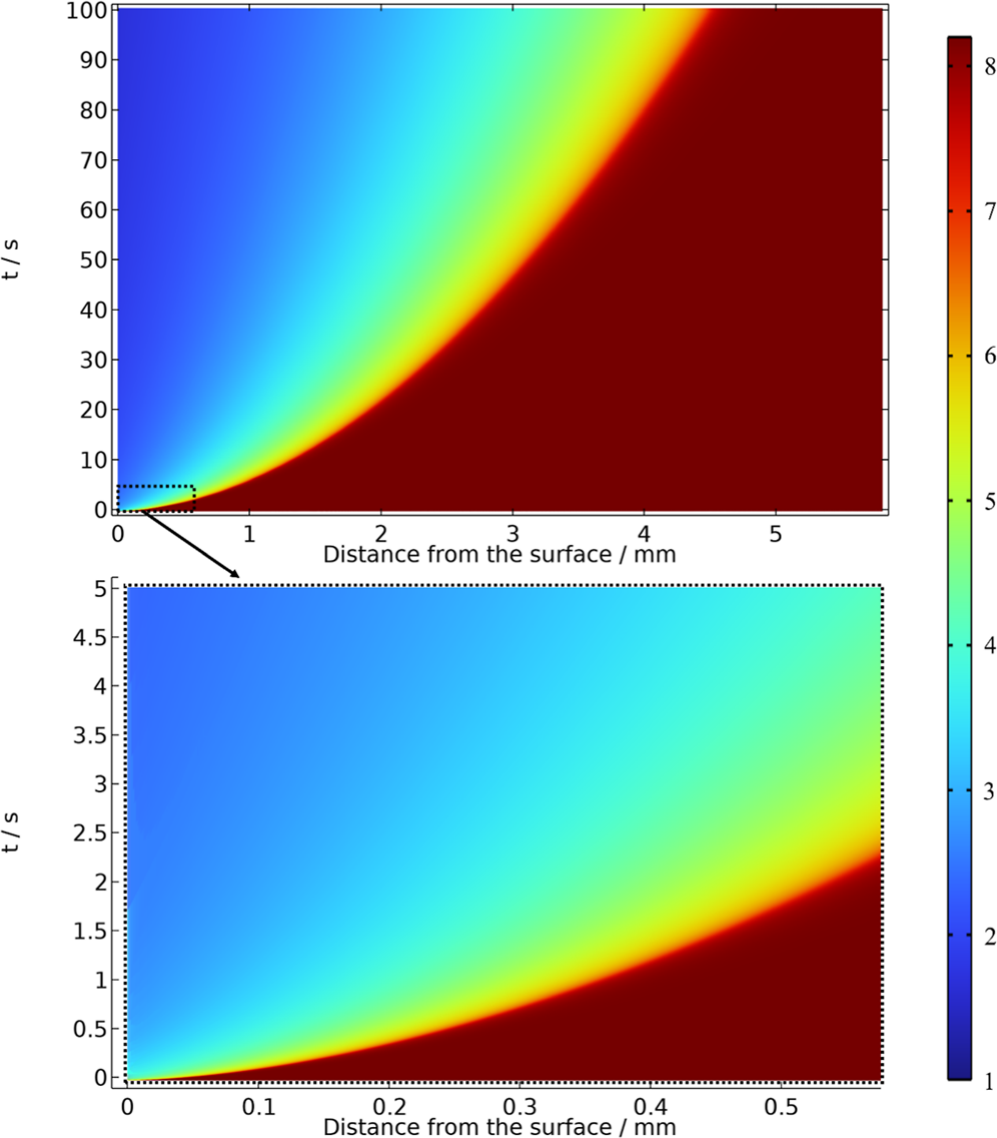
Color scale representation of the spatiotemporal evolution of the pH in the solution in the vicinity of the electrode for a constant current of 0.46 mA cm^–2^ applied at *t* = 0. While the top plate represents the pH changes from 0 to 100 s, the bottom plate represents the first 5 s seconds after polarization.

Just a few seconds after the current was applied, the pH shifts from the bulk values to lower values to 3 (blue scale). In this simulation time scale, the local pH did not reach a steady state, and the depletion layer grew continuously. Obviously, the higher the applied current density, the more acidic the local pH became and the larger the depletion layer was, reaching more than 4 mm at the end of the simulation. The spatiotemporal analysis for the other currents of timeseries in Figure 3 can be found in the Supporting Information section and they bring the similar behavior of Figure 7. Regardless of the current, NSSpH reached the state during the timeseries in which oscillations are observed at least 5 units lower than the bulk pH after 10 s; therefore, the observed behavior agrees with the work of Melle et al.^22^ that concluded oscillations does not emerge during methanol electro-oxidation reaction at pH > 3.5.

Interestingly, after the initial stages of the electro-oxidation process, the interface behaves like those in acidic media, which directly impacts not only the oscillations but also the product distribution and intermediate surface populations. The most analytical techniques employed to follow species in interfaces, such as in situ FTIR and Raman spectroscopy or online mass spectrometry and HPLC, have been performed in a quiescent solution. When these techniques are applied for reactions in neutral media, the NSSpH decrease will reflect the features of acidic media.

## 4 Conclusions

The electrochemical oxidation of methanol and small polyols at Pt in unbuffered solutions with pH near 7 was studied in both quiescent solutions and a rotating disc electrode system. Online mass spectrometry measurement and mathematical modeling allow us to conclude that regardless of the alcohol employed, the interfacial pH considerably decreases during the oxidation process resulting in the following:

1 The appearance of a redox couple at −0.41 V vs Ag/AgCl/KCl that can be addressed to the pair H^+^/H_2_. H_2_ was confirmed by online mass spectrometry, and the oxidation/reduction processes were successfully reproduced by computer simulations, validating the proposed model.
2 The potential oscillations observed under galvanostatic control can be related to alcohol oxidation in acidic media. According to our model, the pH can decrease to 1.68 after 100 s at 0.46 mA cm^–2^. The pH region corresponds to the regions in which oscillation is observed for both methanol and ethylene glycol electro-oxidation reactions. Therefore, the oscillations observed in pH 7 in an unbuffered solution correspond to the process occurring in acidic media due to the pH changes.
3 The mass-transport control avoids the pH decrease due to proton accumulation near the surface and leads to the disappearance of the H^+^/H_2_ pair.

The mathematical modeling employed here can be extended to other reactions that involve coupled proton and electron transfer allowing estimation of NSSpH.

## Supplementary Material



## Notes

Cyclic voltammograms (CV) of Pt in the absence of organic molecules, as well as the CV profile evolution along the cycles for methanol oxidation in both quiescent solutions and under mass-diffusion control; all the galvanostatic timeseries employed to build Table 2; and spatiotemporal evolution of the local pH for the currents 0.130, 0.230, and 0.930 mA cm^–2^ can also be found in this section (PDF)
