# Supplementary material for: Electrochemical Oxidation of Methanol and Small Polyols in Neutral Media: The Effect of the Interfacial pH on Dynamic Instabilities
Source: ACS Omega. 2025 Dec 16;10(51):63455–63. doi: 10.1021/acsomega.5c10287 (PMC12756832; doi:10.1021/acsomega.5c10287)
Supplement: Supplementary file 1 [file ao5c10287_si_001.pdf]

SUPPLEMENTARY FILES OF

ELECTROCHEMICAL OXIDATION OF METHANOL AND SMALL POLYOLS IN NEUTRAL MEDIA: THE EFFECT OF THE INTERFACIAL pH ON DYNAMIC INSTABILITIES

Nayara Gomes dos Santos[a], Evaldo Batista Carneiro-Neto[a], Lauren Moreti[a], Rafael Luiz Romano[b], Fabio Henrique Barros de Lima[b], Ernesto Chaves Pereira[a], Elton Sitta* [a]

[a] Department of Chemistry, Federal University of Sao Carlos, Rod. Washington Luis, km 235, ZIP 13565-905, Sao Carlos, SP, Brazil

[b] São Carlos Institute of Chemistry (IQSC), University of São Paulo (USP), ZIP 13560-970, São Carlos, SP, Brazil

* corresponding author: esitta@ufscar.br

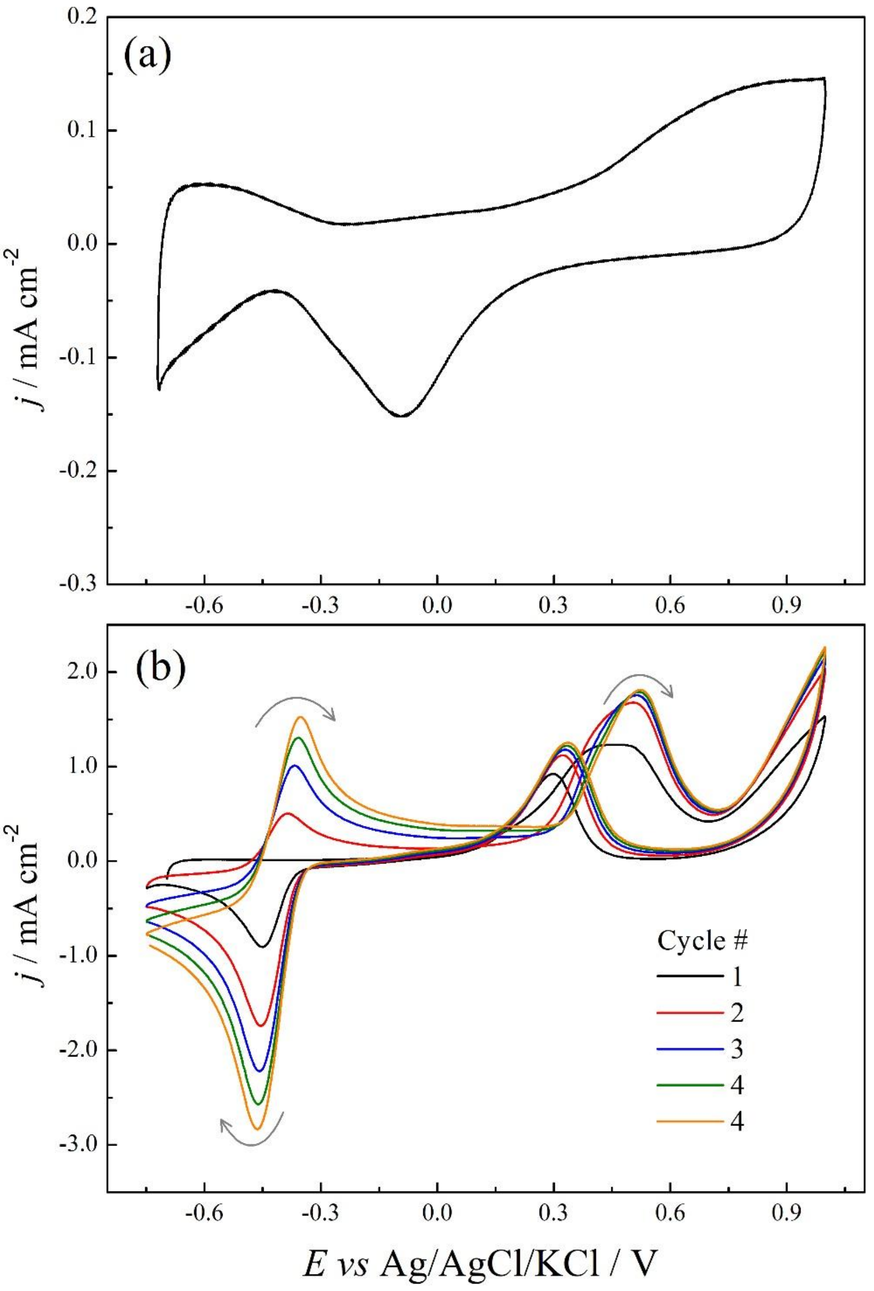

Figure S 1 - Pt cyclic voltammogram at 0.1 V s^{-1} in 0.5 mol L^{-1} Na_2SO_4 in the absence (a) and in the presence of methanol (1.0 mol L^{-1}) (b).

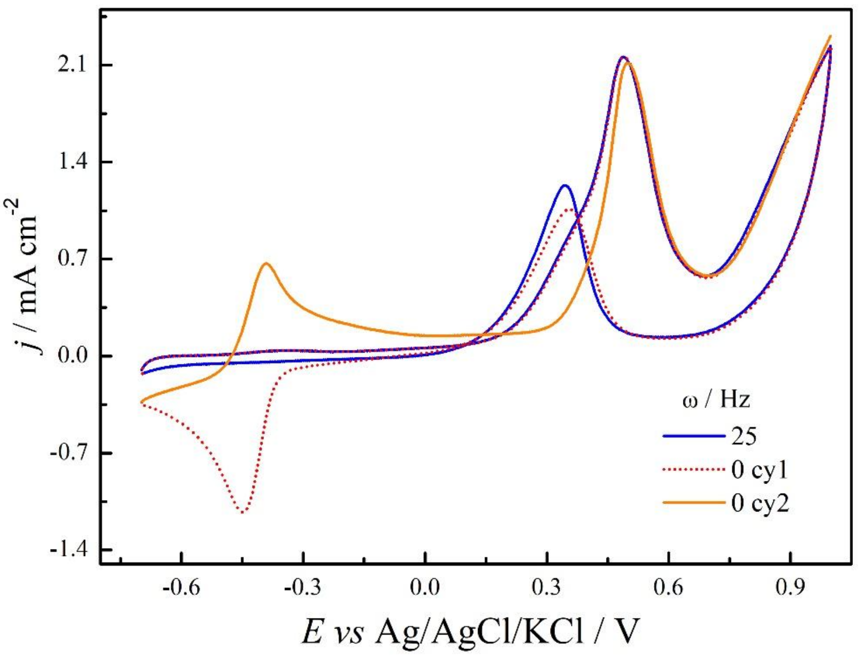

Figure S 2 - Pt cyclic voltammogram at 0.1 V s^{-1} in 0.5 mol $L^{-1}Na_2SO_4$ + 1.0 mol L^{-1} methanol.

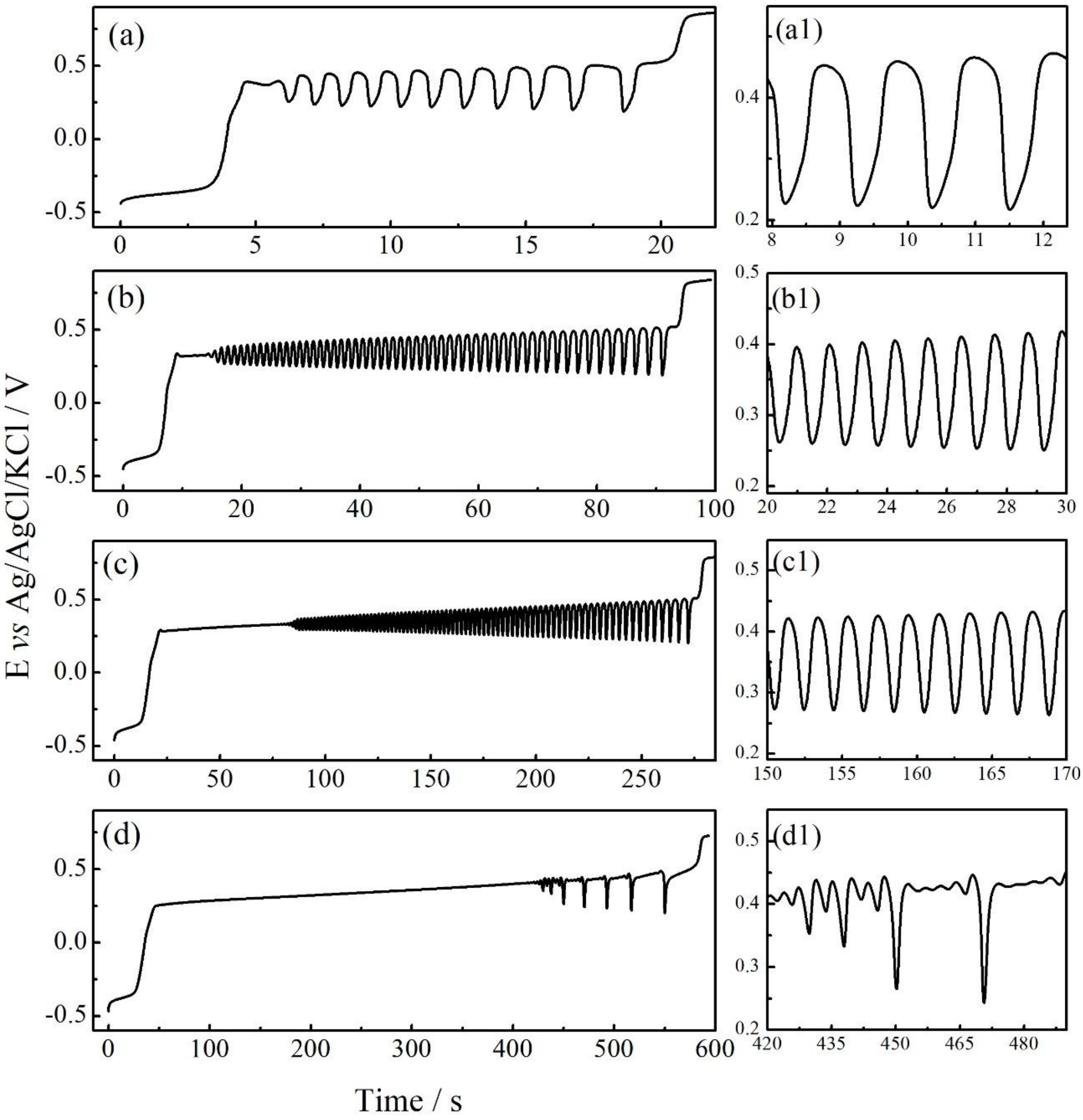

Figure S 3 – Methanol oxidation at constant current of 0.93 (a); 0.46 (b); 0.23 (c), 0.12 mA cm^{-2} (d). The planes a1, b1, c1, and d1 are the same timeseries of a, b c, and d respectively, but with a zoom up.

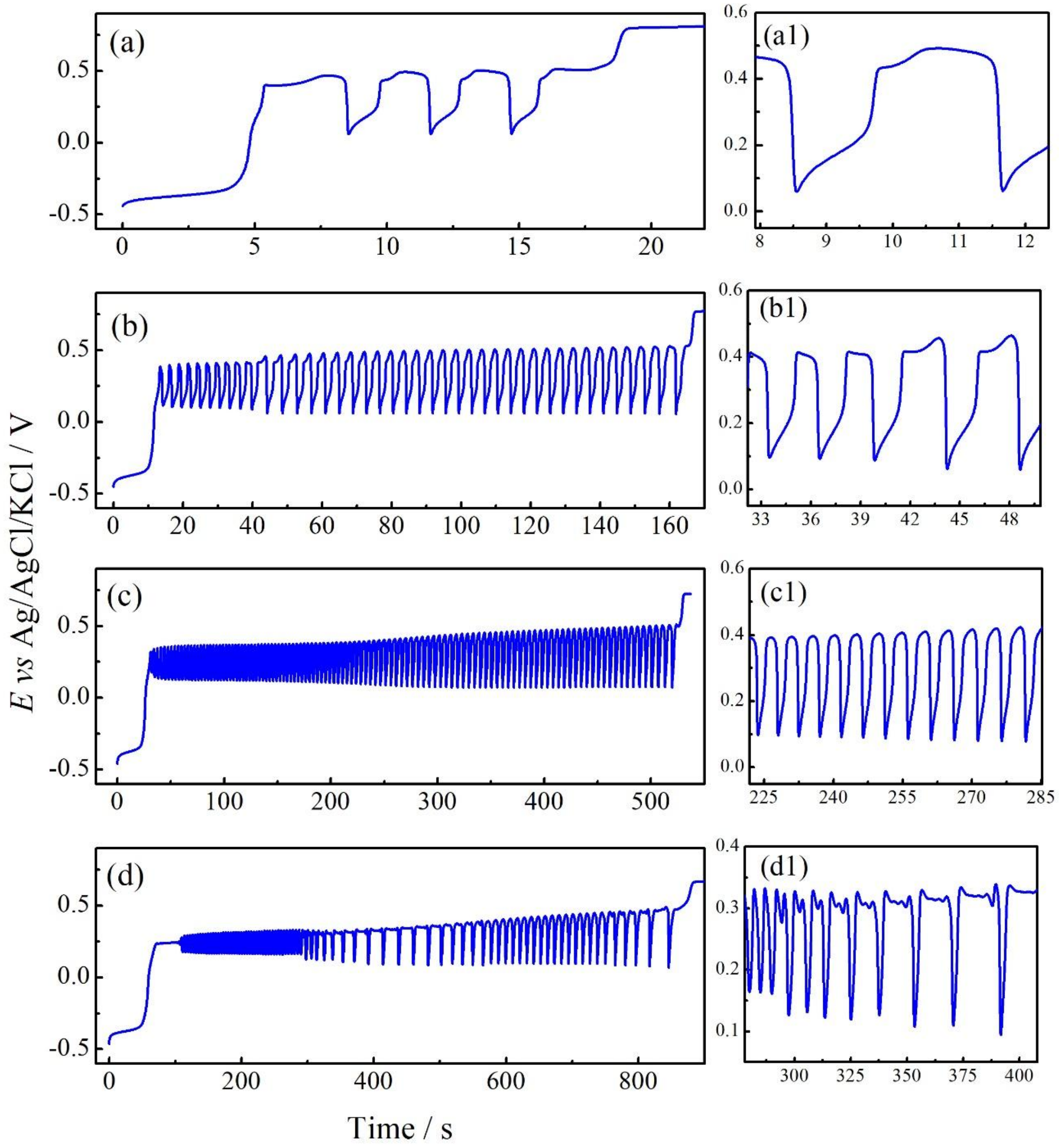

Figure S 4 - Ethylene glycol oxidation at constant current of 0.787 (a); 0.393 (b); 0.197 (c), 0.098 mA cm^{-2} (d). The planes a1, b1, c1, and d1 are the same timeseries of a, b c, and d respectively, but with a zoom up.

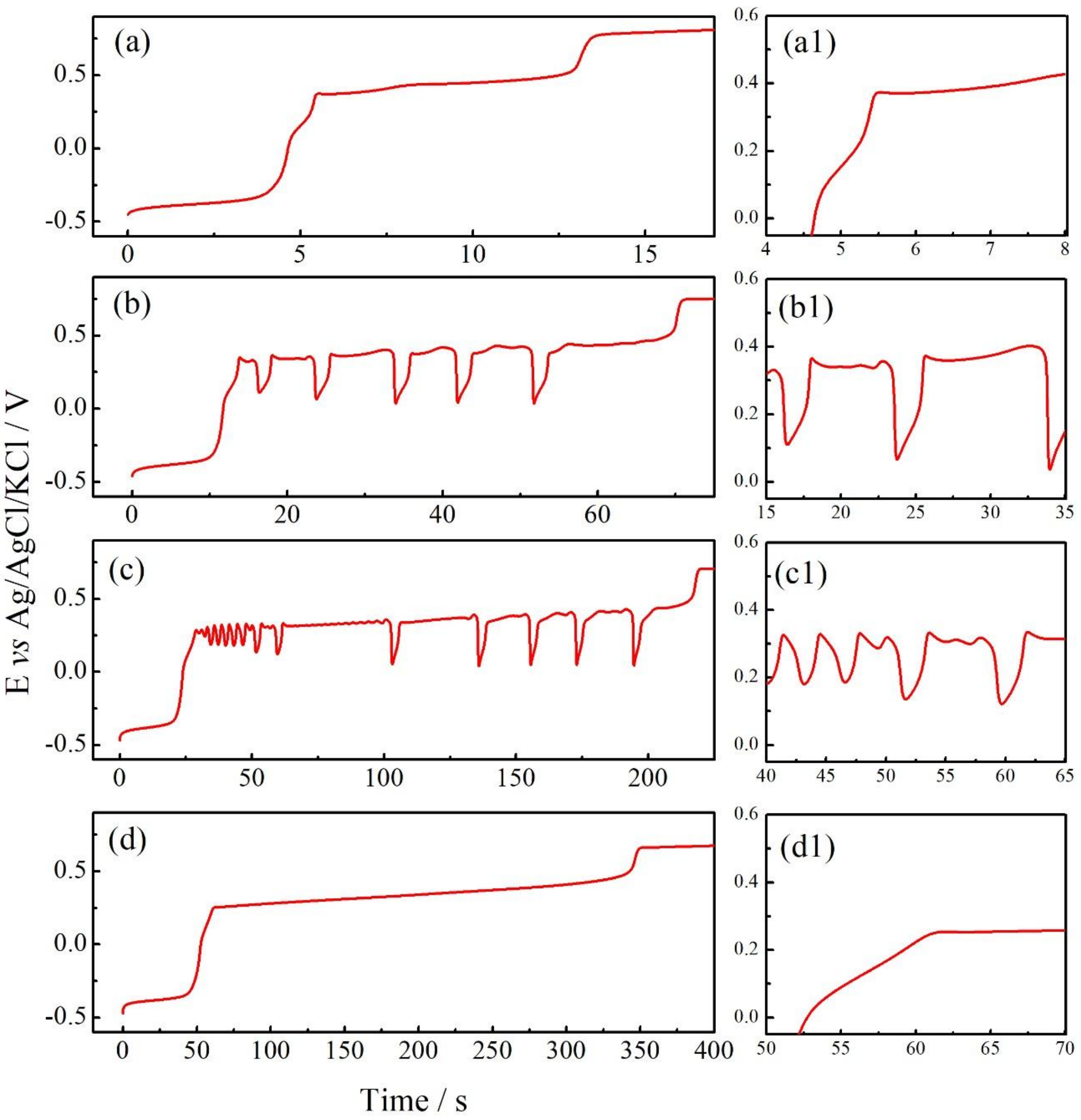

Figure S 5 – Glycerol oxidation at constant current of 0.630 (a); 0.315 (b); 0.157 (c), 0.079 mA cm^{-2} (d). The planes a1, b1, c1, and d1 are the same timeseries of a, b c, and d respectively, but with a zoom up.

Frequency estimation method

$$\omega_n = \frac{1}{t_{E_{max,n}} - t_{E_{max,n-1}}}$$

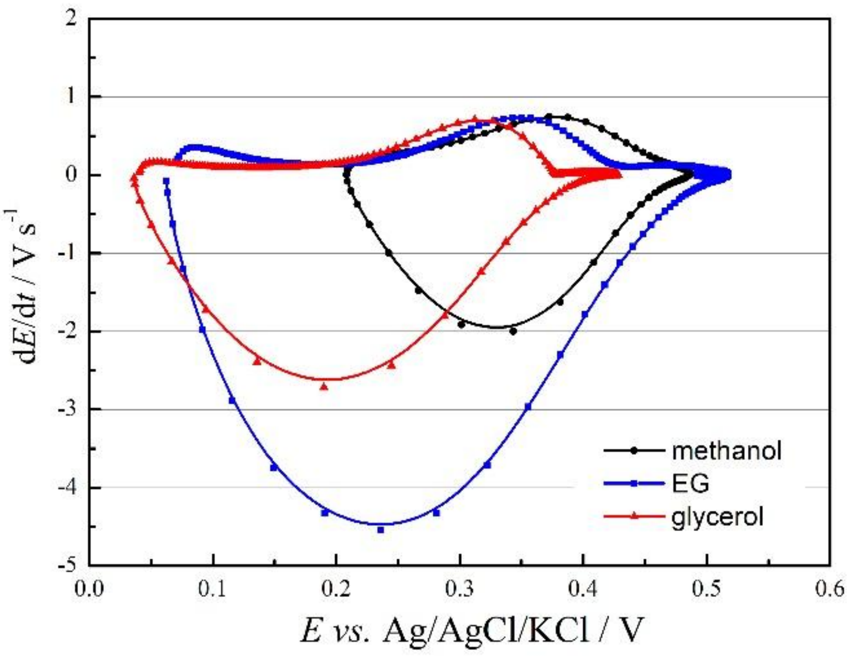

Figure S 6 - Poisoning/Freeing rates (dE/dt) from timeseries.

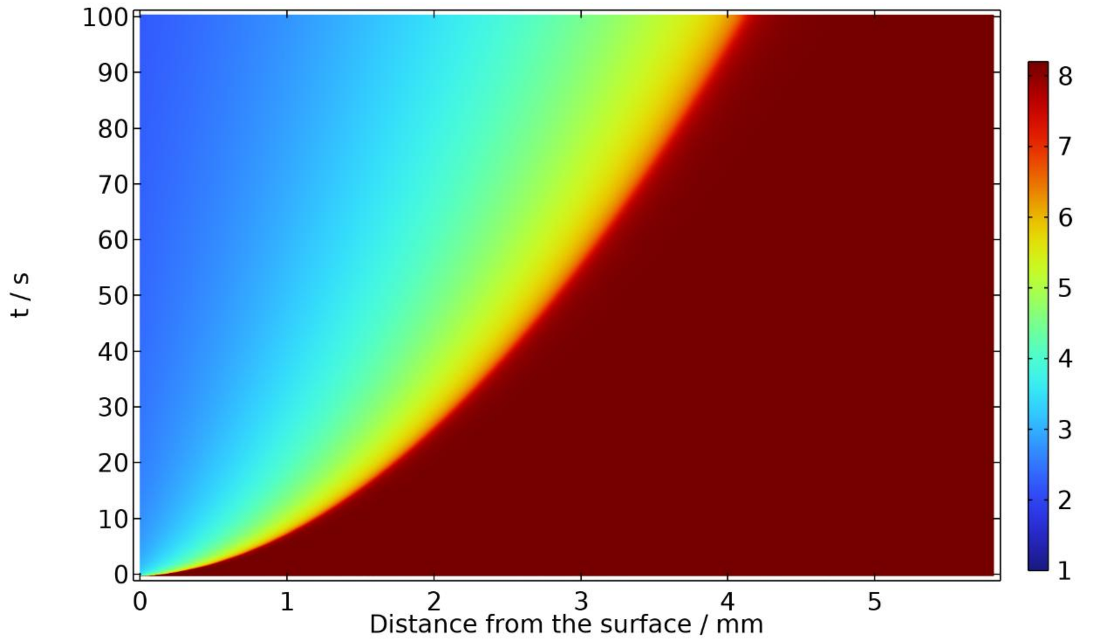

Figure S 7 – Color scale representation of the spatiotemporal evolution of the pH in the solution in the vicinity of the electrode for a constant current of $0.130\ mA\ cm^{-2}$ applied at $t = 0$.

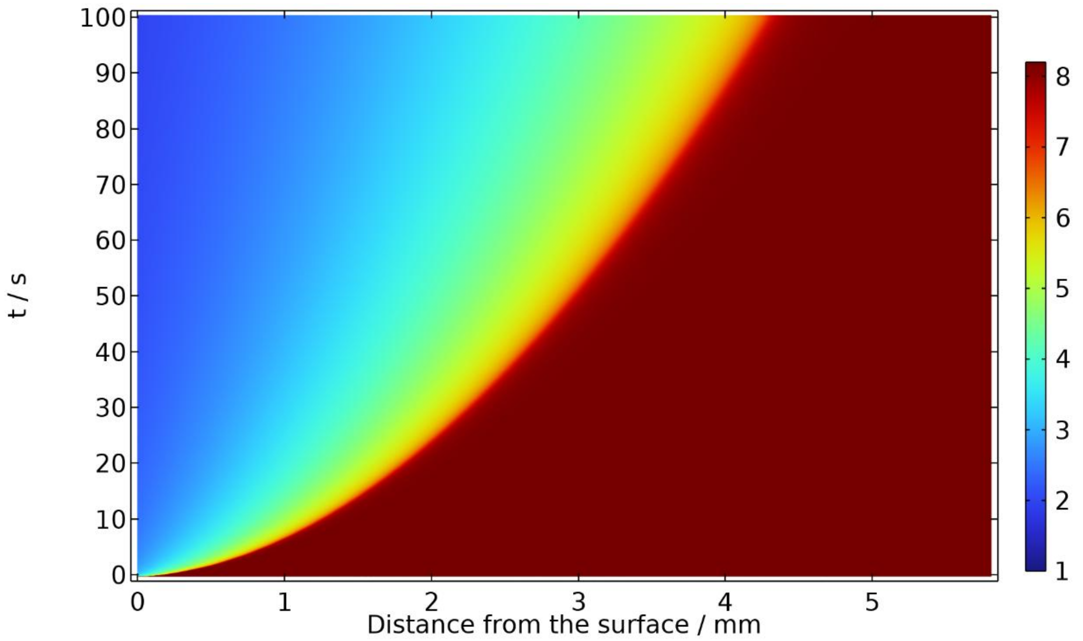

Figure S 8 Color scale representation of the spatiotemporal evolution of the pH in the solution in the vicinity of the electrode for a constant current of $0.230\ mA\ cm^{-2}$ applied at $t = 0$.

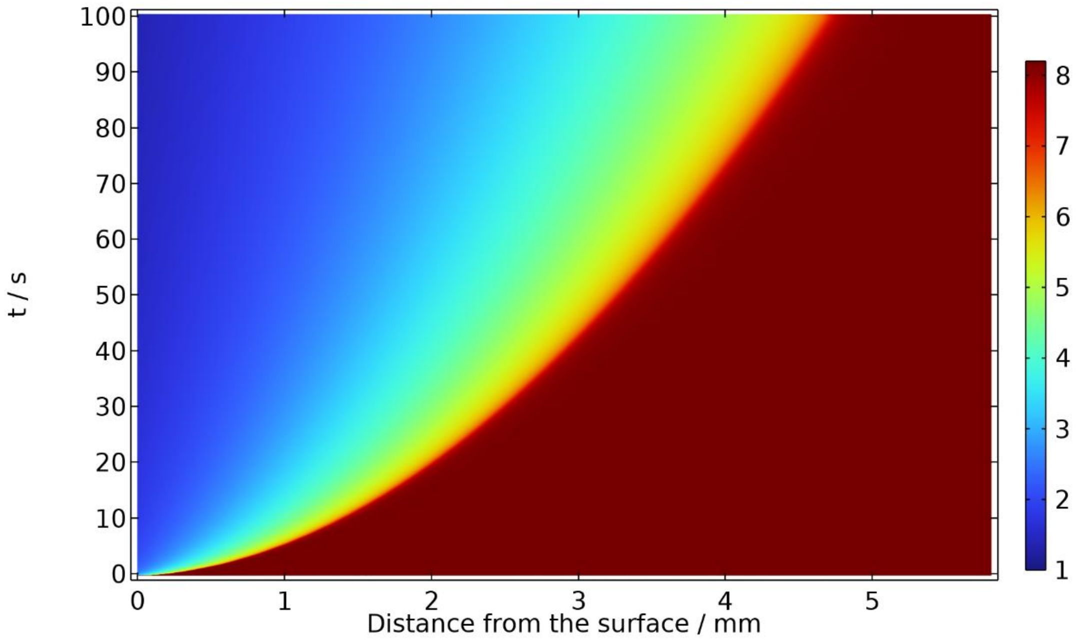

Figure S 9 - Color scale representation of the spatiotemporal evolution of the pH in the solution in the vicinity of the electrode for a constant current of $0.930\ mA\ cm^{-2}$ applied at $t = 0$.
